# Association between Functional MICA-TM and Behcet’s Disease: A Systematic Review and Meta-analysis

**DOI:** 10.1038/srep21033

**Published:** 2016-02-15

**Authors:** Jun Zhang, Dan Liao, Lu Yang, Shengping Hou

**Affiliations:** 1 The First Affiliated Hospital of Chongqing Medical University, Chongqing, China; 2 Chongqing Eye Institute and Chongqing Key Laboratory of Ophthalmology, Chongqing, China

## Abstract

The relationships between polymorphisms of the trans-membrane(TM) region located in the major histocompatibility complex (MHC) class I chain–related gene A (MICA) and Behcet’s disease (BD) have been discussed previously, however, the results were contradictory. In this study, we thoroughly assess whether MICA-TM gene variants are associated with BD by means of a systematic review and meta-analysis. Our study focused on the effects of polymorphisms of MICA-A4, A5, A5.1, A6, and A9 from the included articles. Sixteen previous original publications representing 1,555 BD patients and 2,086 unrelated healthy controls analyzed the association of BD with MICA-TM gene polymorphisms. For the five alleles, MICA-A6 showed a strongly positive correlation with BD patients and could be viewed as an increased risk factor of BD (OR = 2.34, 95%CI: 2.02–2.70). Furthermore, MICA-A4, A5, A5.1, and A9 exhibited negative associations with BD (OR = 0.71, 95%CI: 0.58–0.86; OR = 0.75, 95%CI: 0.63–0.90; OR = 0.63, 95%CI: 0.44–0.91; OR = 0.70, 95%CI: 0.58–0.84, respectively). Our meta-analysis confirmed MICA-A6 could be responsible for BD in three ethnic regions and should probably be treated as a risk factor for BD. MICA-A4, A5, A5.1, and A9 could be regarded as protective factors, especially in the Middle East and East Asia.

Behcet’s disease (BD) is a refractory multi-system inflammatory disease, characterized by four common manifestations, as follows: recurrent genital ulcerations, oral aphthous ulcers, skin lesions and ocular lesions, along with symptoms in the gastrointestinal tract, central nervous system, vascular system, joints, kidneys, and lungs^1^. It has been observed worldwide in many ethnic groups, but most commonly in patients from Japan, China, and Korea, as well as along the Silk Route to the countries of the Mediterranean^2^. Although its etiology and pathogenesis are still undefined, multiple genetic factors and environmental risk factors such as infectious triggers are considered to confer susceptibility to the disease^3^.

Many genes have been reported to be associated with BD, including STAT4, interleukin-23 receptor(IL23R), CD40, and IL17^4^. Up to the present, the HLA-B51 molecule has had the strongest known genetic association with BD in many different ethnic groups^2^,^5^,^6^,^7^. However, whether disease susceptibility is influenced by HLA-B51 itself or by some other genes located around HLA-B in linkage disequilibrium with HLA-B51 remains controversial. Recently, the major histocompatibility complex (MHC) class I chain–related gene A (MICA), a functional gene located between the HLA-B and tumor necrosis factor(TNF) genes on the short arm of human chromosome 6, has been reported to be linked with BD in the trans-membrane (TM) region^1^,^8^,^9^. As a stress-inducible antigen, MICA plays an important role in innate and adaptive immune responses by interacting with the natural killer group 2 member D (NKG2D)-activating receptor of natural killer(NK) cells, CD8 T cells, and γδT cells^10^. Exon 5 in the MICA-TM gene is composed of at least five variable alleles (A4, A5, A5.1, A6, and A9) presenting 4, 5, 6, and 9 triplet repeats of (GCT/AGC)^1^. Polymorphisms of the TM region have been studied to investigate the association with BD in several articles, but the results are still disputed, probably due to the different ethnicities, smaller sample size, and bias in the chosen patients or controls in these works.

In order to better understand the genetic risk of MICA-TM in the relationship with BD, we performed a systematic review and meta-analysis to illuminate this association and determine whether the polymorphisms of MICA-TM conferred susceptibility to BD.

## Results

### General characteristics of studies

**Figure 1:**
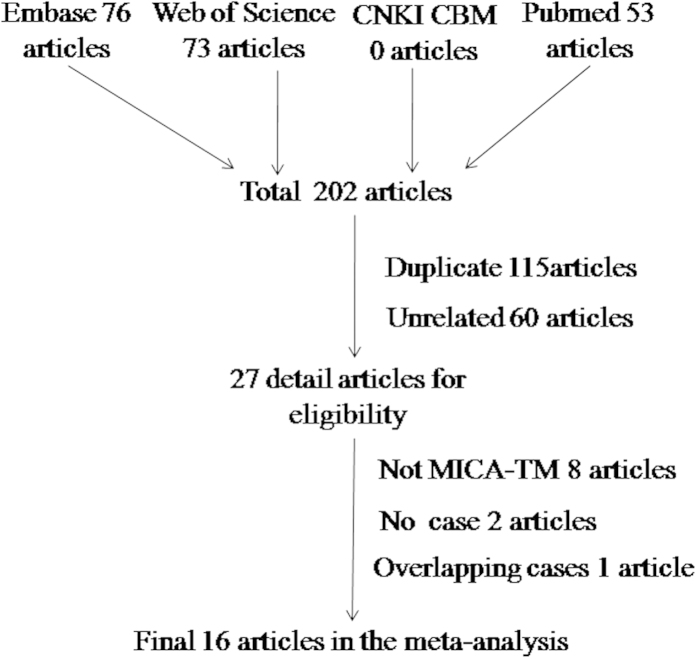
Flow diagram presenting the result of literature searching for meta-analysis.

The selection process of eligible studies is shown in Fig. 1A total of 202 reports were obtained from PubMed, Embase, Web of Science, CBM, and CNKI. Among them, 175 articles were excluded; of which 115 were duplicates and 60 were reviews, meeting reports or articles unrelated to the topic. The other 27 articles were all full-text. Eight articles were excluded because they did not discuss MICA-TM allele variants^11^,^12^,^13^,^14^,^15^,^16^,^17^,^18^, two were excluded because they only compared controls^19^,^20^, and one was excluded because of overlapping patient information with another paper^8^. Finally, 16 publications concerning about the distribution of MICA-TM gene were available for our topic in this meta-analysis^1^,^21^,^22^,^23^,^24^,^25^,^26^,^27^,^28^,^29^,^30^,^31^,^32^,^33^,^34^,^35^. The characteristics of the selected studies are presented in Table 1.

Two articles^1^,^24^ written by the same author had an overlapping sample from Japan, and one article^1^ had more detailed genotyping data, so we decided to exclude the case-control group from Japan in the second article^24^. Therefore, we conducted a meta-analysis that combined 13 studies from 16 articles including 1,304 BD patients and 1,826 unrelated healthy controls for MICA-A4, MICA-A5.1, and MICA-A9; 14 studies including 1,353 BD patients and 1,876 controls for MICA-A5; and 16 studies involving 1,555 BD patients and 2,086 controls for MICA-A6.

### Bias assessment of the included studies

Potential bias assessment of the included studies is presented in Table 2. Among the studies, three articles(18.8%)^1^,^21^,^28^ had bias of ascertainment in the selection of cases, two (12.5%)^30^,^33^ showed possible bias in the population stratification, and the others demonstrated no bias in the selection of cases, controls, genotyping controls, confounding variables, multiple tests, or selective outcome reports.

### Association between MICA-TM allele polymorphisms and BD susceptibility

**Figure 2:**
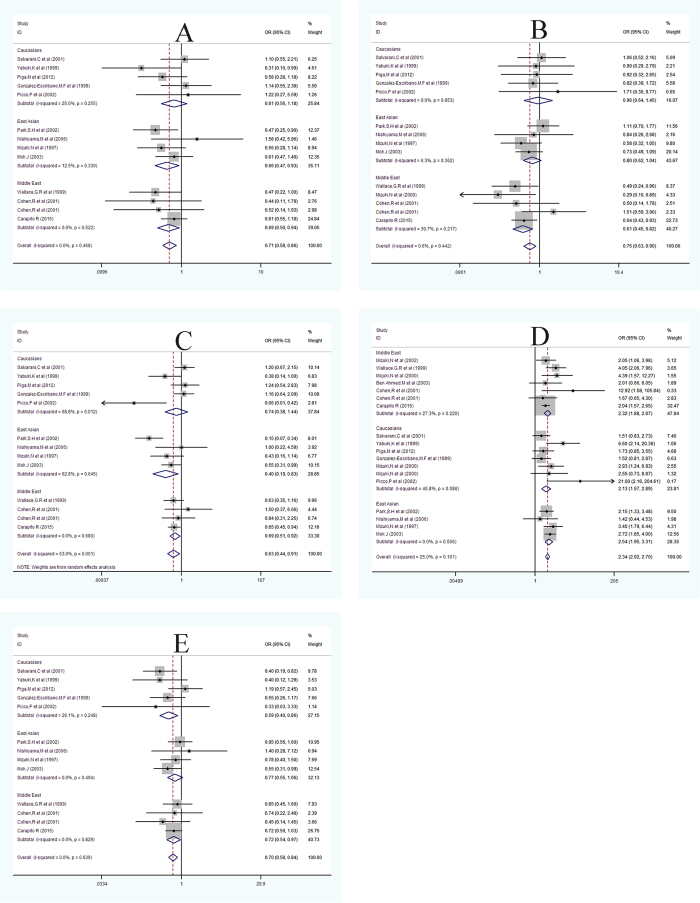
Meta-analysis of the association of MICA-TM polymorphism with Behcet’sdisease (BD). (**A–E**): Forest plot presenting the odds ratios (OR) of BD with MICA- A4, A5, A5.1, A6 and A9 gene in each study, sub- groups based on ethnic group and the pooled results.

Sixteen articles on three ethnicities—namely Middle Eastern, Caucasian and East Asian populations—were incorporated in our meta-analysis. The association between MICA-A4, A5, A5.1, A6, A9, and BD susceptibility was analyzed (Table 3, Fig. 2). We first used STATA 12.0 software to investigate the pooled ORs and heterogeneity. Among the five allele polymorphisms of MICA-TM, the allele MICA-A4, which was studied in 13 studies, was found to be associated with BD. Although only one OR was statistically significant in the relationship with BD, the pooled OR was 0.71(95%CI: 0.58–0.86, Fig. 2A). The MICA-A5 allele was investigated in 14 studies, and only two of the all research had a positive relationship with BD. The pooled OR was 0.75(95%CI: 0.63–0.90, Fig. 2B). The pooled OR of the MICA-A5.1 allele from 13 studies was 0.63(95%CI: 0.44–0.91, Fig. 2C). The MICA-A6 allele was investigated from 18 studies, and 11 of these were related to BD. The pooled OR was 2.34(95%CI: 2.02–2.70, Fig. 2D). The MICA-A9allele, which was reported to show a statistically significant difference in one article, demonstrated significance and the pooled OR was 0.70(95%CI: 0.58–0.84, Fig. 2E).

We then performed a sub-group analysis based on ethnicity. For the MICA-A4allele, statistics derived from three ethnicities (Middle Eastern, Caucasian, and East Asian) indicated an overall I^2^ of 0.0% and ORs of 0.69(95%CI: 0.50–0.94), 0.81(95%CI: 0.56–1.18), and 0.66(95%CI: 0.47–0.93), respectively. For the MICA-A5allele, statistics derived from the three ethnicities showed an overall I^2^ of 0.6% and ORs of 0.61(95%CI: 0.45–0.82), 0.96(95%CI: 0.64–1.45), and 0.80(95%CI: 0.62–1.04), respectively.

For the MICA-A5.1allele, statistics derived from the three ethnicities showed an overall I^2^ of 63.0% and ORs of 0.69(95%CI: 0.51–0.92), 0.74(95%CI: 0.38–1.44), and 0.40(95%CI: 0.19–0.83), respectively. For the MICA-A6allele, statistics derived from the three ethnicities showed an overall I^2^ of 25.0% and ORs of 2.32(95%CI: 1.88–2.87), 2.13(95%CI: 1.57–2.89), and 2.54(95%CI: 1.95–3.31), respectively. Finally, for MICA-A9, the statistics derived from the three ethnicities showed an overall I^2^ of 0.0% and ORs of 0.72(95%CI: 0.54–0.97), 0.59(95%CI: 0.40–0.86), and 0.77(95%CI: 0.55–1.06), respectively.

### Sensitivity analysis

Sensitivity analysis was conducted by the authors to evaluate the effect of each study on the pooled ORs by omitting each study in turn. The pooled ORs were not affected by excluding any study (data not shown).

### Publication bias

A series of Begg’s funnel plots and Egger’s regression tests were applied to detect the publication bias for the five alleles of MICA-TM. Then, five symmetrical funnel plots were depicted for all of the alleles (data not shown). As shown in Table 3, we did not find any obvious publication bias for the association between MICA-TM and BD.

## Discussion

Articles referring to the relationship between MICA-TM polymorphisms and BD have been published over the past 10 years. In this meta-analysis, we included 1,555 BD patients and 2,086 unrelated healthy controls from 16 articles and performed a detailed review related to three ethnicities. The results suggested that the MICA-A6 allele can be treated as an increased risk factor of BD, with a pooled OR of 2.34 while the MICA-A4, A5, A5.1, and A9 alleles can be cautiously viewed as protective factors, with ORs of 0.71, 0.75, 0.63, and 0.70, respectively. Importantly, the MICA-A6 allele was associated with BD in all three ethnic groups. The other alleles were only relevant in Middle Eastern or East Asian patients with BD. In other words, the MICA-A4, A5, A5.1, and A9 allele polymorphism analyzed was not significantly associated with BD in patients from non–Silk Route regions. It could be that the relatively small number of subjects in each study can explain this inconsistent result.

The MICA gene is the nearest neighbor of HLA-B identified to data(only 47kb centromeric) and is by far the most divergent MHC-I known. Similar to the protein fold of MHC class I and homologs, the structure of MICA gene contains long open-reading frames encoding for MHC class I molecules with three distinct extracellular domains(α1, α2 and α3), a transmembrane segment, and a cytoplasmic tail, each encoded by a separate exon^36^. Steinle *et al.*^37^ found that a single amino acid substitution at position 129 in the α2 domain of MICA altered the affinity of binding to the activating natural killer group 2, member D (NKG2D). Zou *et al.*^38^ reported that a nucleotide insertion at the transmembrane region of MICA, resulting a truncated TM region, lead to resist its down-regulation and thereby is functionally relevant in the elimination of virus-infected cells. Whether external region or TM region is more important for the functional role of MICA remains unclear. Further studies are needed to elucidate the exact roles of MICA.

As with classical MHC-I gene, MICA is characterized by its high degree of allele polymorphism which are mostly localized in the exon 2, 3 and 4 (extracellular domains)^39^. Steinle *et al.*^37^ found that MICA*01 and *07 in the α1α2 domain, but not MICA*04, *08 and *16, had a reduced binding affinity with NKG2D and the amino acid substitution of methionine by valine likely affected NKG2D binding indirectly by a conformational change. Additionally, unusual variability in exon 5 presents a microsatellite polymorphism encoding a distinct number of alanine residues in the transmembrane domain corresponding to the microsatellite alleles A4, A5, A5.1, A6, A9^2^. Steinle and co-workers^40^ believed that these changes did not alter the overall hydrophobic character of the molecule, neither affected surface expression of MICA, so that the function of this variability may be questionable. Even though we still did not know whether the extracellular domains of the MICA molecule or the transmembrane region determined the significant function related to the mechanism of immune response, a possible hypothesis have been proposed that a single amino acid insertion/deletion in the MICA-TM region with α-helix leads a net of rotation of about 100° of the extracellular with respect to the cytoplasmic domain^30^. Maybe, this would result in substantial modification of the intermolecular interaction mediated by both the extracellular and cytoplasmic parts of MICA. As a result of this assumption, the pronounced difference in binding affinities of triplet repeats for NKG2D could have significant effects on NK cell activation and the modulation of T-cell response,which could play a role in precipitating or exacerbating autoimmune response.

Yabuki and his colleagues^23^ argued that two hypotheses can account for the primary involvement of the MICA molecule with BD. First, the local immune response *in vivo* is induced after bacterial infection, resulting in stress-induced expression of MICA. Secondly, some bacterial components could have a specific role similar to that of super-antigens in the activation of the MICA molecule. Both hypotheses indicate that the increased MICA-A6 molecule may activate γδT cells, thereby triggering the unusual immune response related with BD.

Although the role of polymorphisms in the TM region of MICA gene is still under debate, the potential correlation between MICA-TM and the development of BD has been investigated in different ethnic groups, including East Asian, Caucasian, and Middle Eastern populations from Japan through to Israel. In our meta-analysis, MICA-A6, the most investigated allele, was found to confer susceptibility to BD with a pooled OR of 2.34 in all three ethnic groups. This result is consistent with those of the original articles included in our study, suggesting that A6 was a common risk gene for BD. Additionally, this gene was a causative risk gene or strongly linked with the true risk gene of BD. The functional or fine-mapping studies are needed to elucidate the exact role of A6 or this region and will helpful for common drug discovery of BD suitable for all ethnic populations. Picco and his colleagues^30^ found that secreted MICA-A6 may provide better steric conditions for ligation, such as bacterial component binding with γδT cells and NK cells that express MICA molecules, thus leading to the onset of BD. The functional correlation between MICA-A4, A5, A5.1, and A9 and BD has not been reported previously. Only a few of our included articles indicated the association of BD with MICA-A4, A5, A5.1, and A9.

The TM region contains most of the hydrophobic amino acids and is mainly expressed in epithelial cells, fibroblasts, endothelial cells, and monocytes^3^,^41^. Nishiyama and his colleagues^25^ suggested that the A4 allele has a high negative correlation with ocular lesions, and the A5 allele has a negative relationship with iridocyclitis in BD patients. Picco and colleagues^30^ argued that MICA-A5.1 seems to play a protective role in BD patients. Furthermore, Park and colleagues^29^ suggested that patients with allele A9 have less severe BD complications than those without allele A9 in terms of uveitis, thrombosis, and neurological and intestinal involvement.

The common opinion that BD shows a strong association with HLA-B51 has been disclosed in relation to several ethnic groups, including East Asian, Caucasian, and Middle Eastern populations. A previous study^32^, together with results related to Spanish^22^, Greek^23^, and Italian^28^ populations, as well as results presented by Mizuki and his colleagues^24^, indicated that the MICA-TM molecule is strongly associated with BD owing to the linkage disequilibrium with HLA-B51. Additionally, previous studies also showed that MICA-A6 is linked with HLA-B52. However, HLA-B52 didn’t associate with Behcet’s disease. These may be explained by the different linkage models in patients and controls for between MICA and HLA-B52. MICA-TM (A9) was found in linkage disequilibrium with HLA-B52 in controls but not in patients with BD^21^. Many BD patients are HLA-B51 negative, but with another Bw4 allele, which is not associated with BD, suggesting that in addition to HLA-B51, there are other gene play important roles in the development of BD.Mizuki and his colleagues^1^ and Park *et al.*^29^ suggested that MICA-TM alleles rather than HLA-B51 play an important role in the development of BD. Especially for HLA-B51-negative patients from Korea, MICA-A6 could be viewed as a meaningful susceptibility biomarker.

A recent study investigated by Hughes *et al.*^18^ suggested that a noncoding variant site(rs116799036), between the HLA-B and MICA gene, was the true source of BD risk factor. This implied that the risk generally ascribed to HLA-B51 was likely not causal in BD. Conversely, the other study reported by Ombrello *et al.*^42^ indicated that HLA-B51 was much more strongly associated with BD than any SNP and conferred significant risk for BD even after controlling for the effect of rs116799036. These contrary results may be explained by population heterogeneity or statistical methodologies. Hughes *et al.*^18^ performed the HLA genotyping at Turkish and Italian populations and used a reference panel of Northern European ancestry for HLA imputation, whereas Ombrello *et al.*^43^ examined HLA genotyping at Turkish population and used a reference panel of mixed European ancestry.

Collectively, the MICA-TM gene appears to be a strong candidate gene for BD based on three main aspects, as follows: its chromosomal localization^23^, its restricted and heat shock–induced expression in epithelial cells^44^, and its predicted immunological function as a ligand of NK cells and γδT cells^10^. Although it has not been determined whether the HLA-B51 gene itself or the nearby MICA-TM gene B is directly localized in the pathogenesis of BD, the possibility must exist that susceptibility or co-susceptibility gene(s) within the genomic sequence region could be implicated in association with BD. Thus, tests with a combination of HLA-B51 and MICA-TM may act as a better genetic marker for BD.

Despite considerable efforts to detect the potential relationship between MICA-TM alleles and BD, some limitations of this meta-analysis need to be mentioned. First, heterogeneity among the ethnic groups was discovered when investigating the association of MICA-TM with BD. However, based on the results of the sensitivity analysis, it is clear that the overall effect was not affected by heterogeneity. Second, the number of patients and controls was relatively small in each included study; therefore, a much larger sample size from different ethnic populations is required for further analysis. Third, since the ethnic origins of patients and controls were not specified in any of the studies, subjects’ ethnicity and different criteria for controls are potential sources of heterogeneity. Finally, the databases from which we selected eligible studies were English and Chinese; thus, a language bias may have been present in our meta-analysis.

In conclusion, our results demonstrated that MICA-A6 probably confers a strong susceptibility to BD in three ethnic regions and could be treated as a risk factor for BD. MICA-A4, A5, A5.1, and A9 could be regarded as protective factors, especially in the Middle East and East Asia. However, these relationships need to be demonstrated from a pathogenic point of view.

## Materials and Methods

### Search strategy

Articles were collected from the following electronic databases: PubMed, Embase, Web of Science, the China Biomedical (CBM) database, and the China National Knowledge Infrastructure (CNKI) database. All studies were carefully selected and were up to date as of May 17, 2015. The following subject headings and key words were used: “Behcet syndrome,” “Behcet’s syndrome,” “Behcets syndrome,” “Behcet disease,” “Behcet’s disease,” or “BD” and “MHC class I chain–related gene A,” “MICA,” or “MIC-A”, without any limitation imposed.

### Study selection

The retrieved articles selected from electronic databases were archived by two reviewers independently by inspecting the title, abstract, and full-text according to specified standards. Any discordance could be solved through discussion and consensus in collaboration with a third author. Included studies in this meta-analysis needed to meet the following criteria: 1) they sought to determine the association between MICA-TM and BD; 2) a detailed number or percent of MICA-TM alleles could be obtained for cases and controls; 3) they were focused on human beings; and 4) they used the case-control approach. The exclusion criteria were as follows: 1) duplication of a previous article; 2) studies that were case reports, reviews or letters; and 3) insufficient data was provided after contacting the corresponding author.

### Data extraction

Data from the selected studies were extracted independently by two reviewers (J.Z. and D.L.). The following contents from each study were collected: name of first author, year of publication, country of cases and controls, ethnicity, characteristics and number of cases and controls, genotyping method, diagnostic criteria for BD, and frequency or percentage of MICA-TM alleles in cases and controls. Two authors carefully checked the collected data and reached agreement on all decisions. For any disagreements that still existed, a third investigator was asked to resolve the issue through discussion.

### Quality assessment

Quality evaluation of the extracted studies was also performed by two authors (J.Z. and D.L.) based on the *HuGENetHandbook*^45^. Six bias assessment items referring to gene-disease association were incorporated in this handbook, including bias in selection of cases, bias in selection of controls, bias in genotyping cases, bias in genotyping controls, bias in population stratification, confounding bias, multiple tests, and selective outcome reports. The quality ascertainment of every item ranged from “Yes” to “No,” while the label “Unclear” was used if there was not enough information to make a determination. A correction and review was carried out by another author (L.Y.) independently if the two authors dissented with each other’s view. Consensus needed to be attained for all labels after discussion.

### Statistical analysis

The systematic checklists and guidelines in the *HuGENetHandbook*^45^ were applied to perform this meta-analysis. The odds ratio (OR) and 95% confidence interval (CI) were calculated and pooled ORs were analyzed for MICA-TM frequency comparison between BD patients and controls. Cochran’s Q statistic was used to assess heterogeneity (p < 0.1, treated as significant level across studies). Moreover, the quantitative I^2^ statistic was used for estimation of inconsistency in our meta-analysis, representing the percentage of the observed variability due to heterogeneity rather than to chance (no heterogeneity, I^2^ = 0–25%; moderate heterogeneity, I^2^ = 25–50%; largeheterogeneity, I^2^ = 50–75%; extreme heterogeneity, I^2^ = 75–100%)^46^. Either the fixed-effect model (I^2^ < 25% and p > 0.1) or random-effect model (I^2^ ≥ 25% and p < 0.1) was applied for the pooled ORs and 95%CIs according to the heterogeneity. We conducted sensitivity analysis to assess the effect of each study on the pooled ORs by omitting each study in turn. Moreover, subgroup analysis was performed to determine the strength of association of different ethnicities. Publication bias was also checked by Begg’s funnel plots^47^ and Egger’s regression test^48^. STATA12.0 software (StataCorp LP, College Station, Texas, USA) was used to carry out statistical analysis. A significant difference was estimated under the level of 0.05 (a two-tailed p value) except for the Q statistic. All results had to be validated by two authors (J.Z. and D.L.) independently.

## Additional Information

**How to cite this article**: Zhang, J. *et al.* Association between Functional MICA-TM and Behcet’s Disease: A Systematic Review and Meta-analysis. *Sci. Rep.* **6**, 21033; doi: 10.1038/srep21033 (2016).

## Figures and Tables

**Table 1:** The general characteristic information of included studies.

Year(Ref)	First author	Country	Mean age(Y) of BD	Mean age(Y) of control	%Male of BD	%Male of control	Typing technique	Diagnostic criteria	Ethnicity	Study design
2002	Mizuki *et al.*	Iran	NA	NA	NA	NA	Genotyping	I.C.B.D	Middle East	Case-control, ethnic- age-,matched
2001	Salvarani *et al.*	Italy	31.0 ± 12.0	NA	69.5	NA	Genotyping	I.C.B.D	Caucasian	Case-control, ethnic- matched
1999	Yabuki *et al.*	Greece	37.5 ± 10.6	36.5 ± 12.6	71.1	52.5	Genotyping	I.C.B.D	Caucasian	Case-control, ethnic- age-,matched
2002	Park *et al.*	Korea	41.2	NA	NA	NA	Genotyping	I.C.B.D	East Asian	Case-control, ethnic- matched
2012	Piga *et al.*	Italy	NA	NA	NA	NA	Genotyping	I.C.B.D	Caucasian	Case-control, ethnic- matched
1999	Gonzalez *et al.*	Spain	NA	NA	NA	NA	Genotyping	NA	Caucasian	Case-control, ethnic- matched
2000	Mizuki *et al.*	Greece Italy	NA	NA	NA	NA	Genotyping	I.C.B.D	Caucasian/Caucasian	Case-control, ethnic- age-,matched
1999	Wallace *et al.*	Jordan	32.2	NA	80	NA	Genotyping	I.C.B.D	Middle East	Case-control, ethnic- matched
2002	Picco *et al.*	Italy	NA	NA	NA	NA	Genotyping	I.C.B.D	Caucasian	Case-control, ethnic- matched
2000	Mizuki *et al.*	Jordan	26.6	NA	71	NA	Genotyping	I.C.B.D	Middle East	Case-control, ethnic- age-,matched
2006	Nishiyama *et al.*	Japan	47.8 ± 13.7	40.2 ± 13.7	78.2	69.6	Genotyping	J.C.B.D	East Asian	Case-control, ethnic- matched
1997	Mizuki *et al.*	Japan	NA	NA	NA	NA	Genotyping	J.C.B.D	East Asian	Case-control, ethnic- sex-,matched
2003	Ben *et al.*	Tunisia	NA	NA	68.3	NA	Genotyping	I.C.B.D	Middle East	Case-control, ethnic- age-,sex-,matched
2015	Carapito *et al.*	Iran	NA	NA	NA	NA	Genotyping	I.C.B.D	Middle East	Case-control, ethnic- age-, sex-,matched
2003	Mok *et al.*	Korea	NA	NA	NA	NA	Genotyping	I.C.B.D	East Asian	Case-control, ethnic- matched
2001	Cohen *et al.*	Israel(Ara) Israel(NAJ)	NA	NA	NA	NA	Genotyping	I.C.B.D	Middle East/Middle East	Case-control, ethnic- matched

NA, Not Available; I.C.B.D, International Criteria for classification of BD; J.C.B.D, Japanese diagnostic Criteria of BD.

**Table 2:** Assessment of potential bias in included studies.

Year	First author	Bias in selection of cases	Bias in selection of controls	Bias in genotyping controls	Bias in population stratification	Confounding bias	Multiple test and Selective outcome reports
2002	Mizuki *et al.*	NO	NO	NO	NO	NO	NO
2001	Salvarani *et al.*	Yes	NO	NO	NO	NO	NO
1999	Yabuki *et al.*	NO	NO	NO	NO	NO	NO
2002	Park *et al.*	NO	NO	NO	NO	NO	NO
2012	Piga *et al.*	NO	NO	NO	NO	NO	NO
1999	Gonzalez *et al.*	NO	NO	NO	NO	NO	NO
2000	Mizuki *et al.*	NO	NO	NO	NO	NO	NO
1999	Wallace *et al.*	Yes	NO	NO	NO	NO	NO
2002	Picco *et al.*	NO	NO	NO	Unclear	NO	NO
2000	Mizuki *et al.*	NO	NO	NO	NO	NO	NO
2006	Nishiyama *et al.*	NO	NO	NO	NO	NO	NO
1997	Mizuki *et al.*	Yes	NO	NO	NO	NO	NO
2003	Ben *et al.*	NO	NO	NO	NO	NO	NO
2001	Cohen *et al.*	NO	NO	NO	NO	NO	NO
2015	Carapito *et al.*	NO	NO	NO	NO	NO	NO
2003	Mok *et al.*	NO	NO	NO	Unclear	NO	NO

**Table 3:** Association between MICA-TM polymorphism and BD.

Allele	Number of publication	Test of association			Test of heterogeneity		Publication bias	
		OR	95% CI	Model	I^2^(%)	P value	Begg’s test	Egger’s test
A4	13	0.71	(0.58,0.86)	F	0.0	0.468	0.951	0.579
A5	14	0.75	(0.63,0.90)	F	0.6	0.442	0.827	0.729
A5.1	13	0.63	(0.44,0.91)	R	63.0	<0.01	0.583	0.461
A6	18	2.34	(2.02,2.70)	F	25.0	0.161	0.041	0.075
A9	13	0.70	(0.58,0.84)	F	0.0	0.639	0.669	0.468

F: Fixed effect model; R: Random effect model.
